# Role of Oxidative Stress in Ethanol-induced Neurotoxicity in the Developing Cerebellum

**Published:** 2012

**Authors:** Azam Ramezani, Iran Goudarzi, Taghi Lashkarboluki, Mohammad Taghi Ghorbanian, Kataneh Abrari, Mahmoudi Elahdadi Salmani

**Affiliations:** 1 *Faculty of Biology, Damghan University, Damghan, Iran*

**Keywords:** Cerebellum, Ethanol, Oxidative stress, Purkinje cell, Rat

## Abstract

**Objective(s):**The purpose of this study was to investigate the role of oxidative stress in Purkinje cell neurotoxicity of ethanol-treated rat.

**Materials and Methods:**Male rat pups 4-day-old was used in this study. Ethanol was administered to rat pups at a dose of 6 g/kg from postnatal days (PDs) 4 to 5. Pups were killed 90 min after the second alcohol treatment on PD 5 by decapitation and the brain was immediately removed. The cerebellum was dissected for analyzing the oxidative stress parameters and histological study. The activities of several antioxidant enzymes including superoxide dismutase (SOD), catalase (CAT) and glutathione peroxidase (GPx) in vermis of cerebellum were assayed. Thiobarbituric acid reactive substances (TBARS) levels were also measured as a marker of lipid peroxidation.

**Results:**Administration of ethanol significantly increased TBARS levels in the cerebellum compared to control pups (*P*< 0.01). The treated pups with ethanol exhibited a marked decrease in the GPx activity (*P*< 0.01) whereas, in spite of decrease in the activities of SOD and CAT, when compared to control, there were not significant differences. The spherical cell bodies of Purkinje cells in control rats are aligned nicely between the granular and molecular layers. In ethanol treated pups, Purkinje cells scattered within the Purkinje cell layer and shrinkage of the cell somata is seen.

**Conclusion:**The results of the present work demonstrated that ethanol exposure during the vulnerable window could increase TBARS levels (lipid peroxidation) and decrease GPx levels in pup's cerebellum. Also, the results confirmed ethanol-induced microencephaly, cerebellar Purkinje cell loss. These findings suggest that Purkinje cell loss is, in part through decrease in the activity of GPx and increase of lipid peroxidation in the rat cerebellum.

## Introduction

Prenatal ethanol exposure in the human, via maternal consumption of alcoholic beverages during pregnancy, can produce a wide range of toxic effects, including teratogenicity and lethality, manifesting as spontaneous abortion or stillbirth (1). In the human, ethanol teratogenicity can manifest as the fetal alcohol syndrome (FAS), which consists of the following principal features: central nervous system (CNS) dysfunction, growth deficiency, and a characteristic cluster of facial abnormalities in the offspring (2). Of these three principal features, CNS dysfunction, manifesting as persistent intellectual, behavioral, and neurological deficits, is probably the most debilitating for the individual with FAS. Associated with this CNS dysfunction is permanent neuronal cell loss in target brain regions, including the hippocampus and cerebellum, as demonstrated in experimental animal models of ethanol CNS teratogenicity (3-6).

Cerebellar Purkinje cells are the major output neurons from the cerebellar cortex and are sensitive to various neurotoxic agents and diseases (7). Moreover, this well-organized neuronal population is particularly vulnerable to developmental alcohol-induced damage, such as neuronal loss (death), compared with other neuronal populations in the developing brain (8, 9). This unique feature of increased sensitivity to alcohol’s deleterious effect makes cerebellar Purkinje cells an effective and valuable model system to evaluate the teratogenic effect of alcohol. Importantly, alcohol exposure during the period of extensive connectivity among Purkinje and other neurons, either from postnatal days (PD) 4-6 (10-13) or only on PD 4 (5), results in a significant and permanent loss of Purkinje cells. 

Many mechanisms have been implicated as contributors to developmental alcohol-induced brain damage (8, 14-16). Recently, oxidative stress mediated apoptosis has received much attention in the search for underlying mechanisms (17-21). In several organ systems, including the brain, excessive ethanol exposure can result in increased production of reactive oxygen species (ROS), including superoxide radical anion, hydroxyl radical and hydrogen peroxide, and/or suppression of antioxidant defense mechanisms that normally inactivate ROS, including superoxide dismutase, glutathione and glutathione peroxidase, thereby resulting in oxidative stress (22). Ramachandran *et al* (2003) reported that alcohol treatment led to apoptotic death in primary cortical neuron cultures as measured by increased release of cytochrome c from mitochondria and increased caspase-3 activity and that such apoptotic death was closely related to markers associated with oxidative stress (e.g., malondialdehyde and 4-hydroxynonenal) (23). If oxidative stress does play a central role in alcohol induced damage, then antioxidants should be effective in ameliorating these effects. Moreland *et al* (2002) reported that coadministration of alcohol and the bioflavonoid silymarin to pregnant rats mitigated the detrimental effect of alcohol on the development of the corpus callosum (24). A recent report by Cohen-Kerem and Koren (2003) provided a brief summary of preclinical findings with regard to the protective effects of antioxidants on alcohol-induced teratogenicity (22). Using this Purkinje cell model, Heaton *et al* (2000) demonstrated that neonatal alcohol exposure on postnatal days (PDs) 4 and 5 significantly reduced the Purkinje cell numbers in lobule I of the cerebellum and that such deficits were attenuated by pretreatment and cotreatment with vitamin E (25). Therefore, researcher reports have suggested that oxidative stress is a potential mechanism for alcohol induced injury and that supplementation with antioxidants can ameliorate alcohol-induced damage. However, Edwards *et al *(2002) demonstrated that two known antioxidants, melatonin and U83836E, weren’t effective in blocking the expected alcohol-induced cerebellar Purkinje cell loss in neonatal rat pups (26). Also, Pierce *et al* (2006) reported that ethanol exposure on postnatal day 4 (6.0 g/kg) reduced the number of Purkinje neurons in the cerebellum and Concurrent treatment with antioxidant did not protect the Purkinje neurons from ethanol-related cell loss (27). Thus, with regard to discrepancy of oxidative stress role in Purkinje cell toxicity, the objective of the present study was to investigate the role of oxidative stress in alcohol induced neurotoxicity in cerebellar Purkinje cells of neonate rats.

## Materials and Methods

The experimental protocol was approved by the Research and Ethics Committee of Damghan University. Male rat pups 4-day-old derived from timed matting of adult *Sprague Dawley* rats served as subjects in this study. Animals were kept under standard laboratory conditions with a 12 hr light/dark cycle and *ad libitum* food and water throughout the experiments. 

Pups divided by control and ethanol groups with 8 pups for each group. Alcohol was administered to ethanol group at a dose of 6 g/kg body mass of 35% ethanol solution by i.p. injection in two consecutive injections from PD 4 to 5. Pups were killed 90 min after the second alcohol treatment on PD 5. The second group served as untreated controls and received a daily intraperitoneal (i.p.) injection of saline (0.9% w/v, administered in equal volumes as ethanol-treated rats) in two consecutive injections from PD 4 to 5. 

All blood samples were taken on PD 5 at 90 min following the second injection of the ethanol. Twenty microliters of blood was taken from each subject by producing a small nick to the tip of the tail. The samples were immediately placed into individual glass vials containing 200 ml of a cocktail composed of 0.6 N perchloric acid and 4 mM propanol in double distilled water, tightly fastened with a septum sealed lid, and stored at room temperature until analysis by head space gas chromatography (Model 3900, Varian, Palo Alto, CA) (28). 

Animals were killed by decapitation in PD 5 and the brain was immediately removed and the cerebellum was dissected for analyzing the oxidative stress parameters and histological examination. The other part of tissue samples was homogenized in cold 50 mM sodium phosphate buffer (pH 7.0) containing 0.1 mM EDTA to give 5% homogenate (w/v). The homogenates were then centrifuged at 6000×g for 10 min at 4 °C to remove nuclei and debris. The supernatants were separated, aliquoted, and stored at -80 °C until analysis (29).

Thiobarbituric acid-reactive substances (TBA-RS) were measured according to Ohkawa *et al* (1979). Briefly, to glass tubes were added, in order of appearance: 500 μl of sample; 50 μl of sodium dodecyl sulfate 8.1%; 1500 μl of 20% acetic acid in aqueous solution (v/v) pH 3.5; 1500 μl of 0.8 % thiobarbituric acid; and 700 μl of distilled water. The mixture was vortexed and the reaction was carried out in a boiling water bath for 1 hr. The mixture was allowed to cool on water for 5 min and was centrifuged at 750 g for 10 min. The resulting pink stained TBA-RS were determined in a spectrophotometer at 532 nm. TBA-RS were calculated as nmol/mg protein. A calibration curve was performed using 1, 1, 3, 3-tetramethoxypropane as a standard. TBA-RS were represented as nmol TBA-RS/mg protein (30).

Total SOD activity was assayed according to Becana *et al* (1986) following the inhibition of the photochemical reduction of nitroblue tetrazolium (NBT) (31). The reaction mixture contained 50 mM Na-phosphate (pH 7.8), 0.1 mM EDTA, 14.3 mM methionine, 82.5 µM NBT and 2.2 µM riboflavin. The reaction was initiate by placing the test tubes under 15 W fluorescent lamps. The reaction was terminated after 10 min by removing the reaction tubes from the light source. Non-illuminated and illuminated reactions without supernant served as calibration standards. The reaction products were measured at 560 nm. One unit of SOD (U) was defined as the amount of enzyme that produced a 50% inhibition of NBT reduction under assay condition. 

CAT activity was assayed by the method of Aebi (1984) using spectrophotometery (32). This method is based on the disappearance of H_2_O_2_ at 240 nm in a reaction medium containing 20 mM H_2_O_2_, 0.1% Triton X-100 and 10 mM potassium phosphate buffer pH 7.0. CAT activity is represented as absorption change in time unit (1 min) per mg protein. 

GPx activity was measured according to the method of Wendel (1981) using tert-butyl hydroperoxide as substrate (33). NADPH disappearance was monitored at 340 nm using a spectrophotometer. The reaction medium contained 2 mM glutathione, 0.15 U/ml glutathione reductase, 0.4 mM azide, 0.5 mM tert-butyl hydroperoxide and 0.1 mM NADPH. GPx activity is represented as absorption change in time unit (1 min) per mg protein. 

Protein concentration was determined in cereberal cortex homogenates using bovine serum albumin as a standard (34).

For histological study, one halve of cerebellum immersed in 10% buffered paraformaldehyde for a week. Then, the cerebella were cryoprotected with 30% sucrose in phosphate buffered saline, pH 7.4 (PBS) overnight. Mid-vermal sections of 5 micron thickness were prepared by cryostat sectioning (Leica, Germany). For determination of Purkinje cell loss following ethanol exposure, monoclonal antibody to calbindin D28k (C9848, Sigma-aldrich) was used to selectively identify Purkinje cells (35). Immunofluorescence for the calbindin D-28 k-positive cells in the cerebellar vermis was performed. 

Tissue sections were washed in PBS and then the endogenous peroxidase was blocked by incubation for 30 min at room temperature with 0.1% H_2_O_2_ in PBS containing 0.3% Triton X-100. Sections were washed three times for 5 min each in PBS. The sections were then incubated at 4 °C, overnight with mouse anti-cabindin D-28 k antibody (1:1000; Sigma Chemical Co.). Sections were washed three times for 10 min each in PBS. The sections were next incubated for 2 hr with fluorescein isothiocyanate (FITC)-conjugated goat anti-mouse secondary antibody (Chemicon-AP124F). The sections were then mounted on gelatin-coated glass slides and the coverslips were mounted using fluorescent mounting medium. The slides of the fluorescent images were captured using a fluorescent microscope (Nikon). 

***Statistics analysis***

Student's t test was used to compare TBARS levels, the activities of SOD, CAT, GPx and cerebellum to body weight ratio among groups. In each test, the data were expressed as the mean ± SEM and *P*< 0.05 was accepted as statistically significant. 

## Results

Blood ethanol concentration (BEC) at 90 minute following the second ethanol exposure was 322±29 mg/dl for the ethanol group (n= 7). 

**Figure 1:**
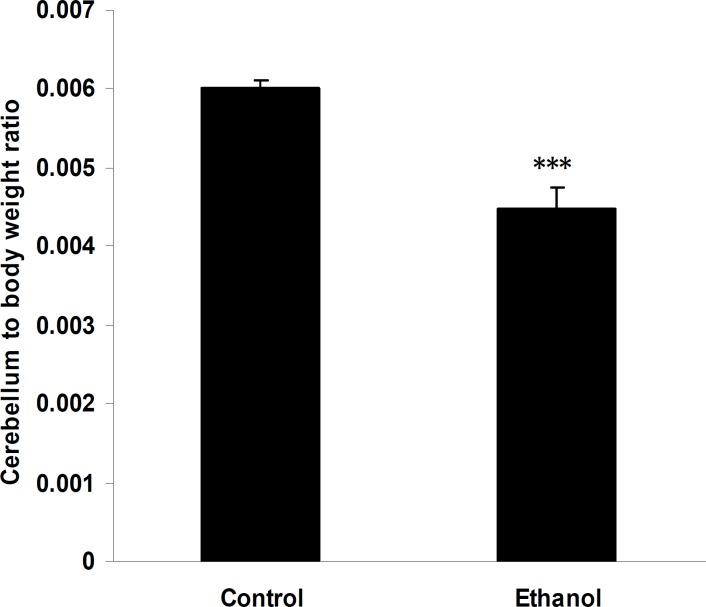
Effects of ethanol on the average of cerebellum to body weight ratio on PND 5. Animals were given ethanol or NaCl 0.9% from PDs 4 to 5 and killed 90 min after the last dose. Data are mean±SEM (n= 8)

The cerebellum to body weight ratio was calculated by dividing the cerebellum weight by the body weight of pup at sacrifice and multiplying by 100. There was a significant difference between the ethanol and control groups in cerebellum to weight ratio (Figure 1, *P* ≤0.001, n= 8); the cerebellum to body weight ratio was higher for the pups in control group (0.006±0.0001) relative to that of the pups in the ethanol group (0.004±0.0002). 

**Figure 2:**
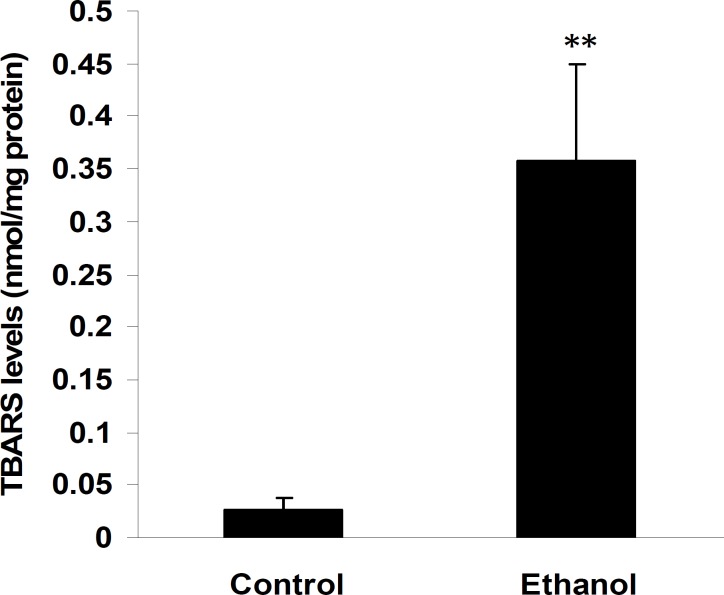
TBARS levels in the cerebellum of control and pups exposed to ethanol (6 g/kg) or a vehicle (NaCl 0.9%) intraperitoneal injection. Administration of ethanol significantly increased TBARS levels as compared to control pups. Values were expressed as mean ± SEM (n= 7 per group)

**Figure 3:**
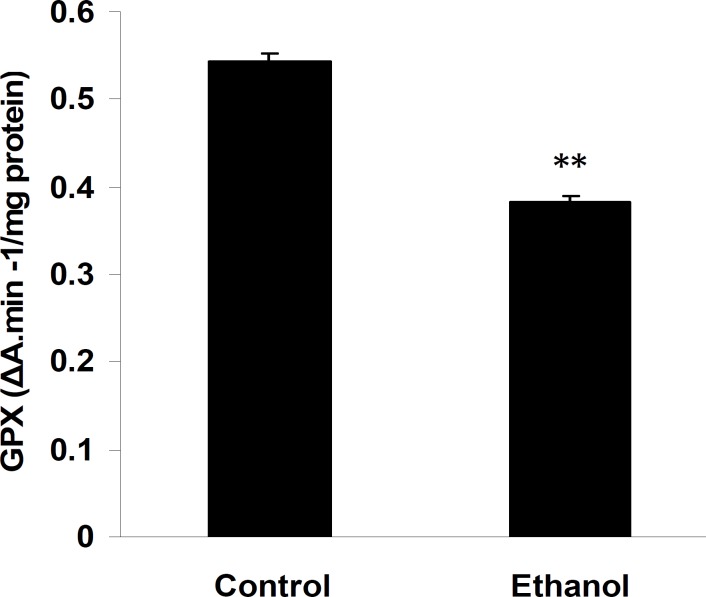
Glutathione peroxidase activity in the cerebellum of control and pups exposed to ethanol (6 g/kg) or a vehicle (NaCl 0.9%) intraperitoneal injection. Ethanol significantly diminished the glutathione peroxidase activity in the cerebellum of animals. Results are presented as mean ± SEM (n= 7 per group). ΔA: Absorption change.

**Figure 4:**
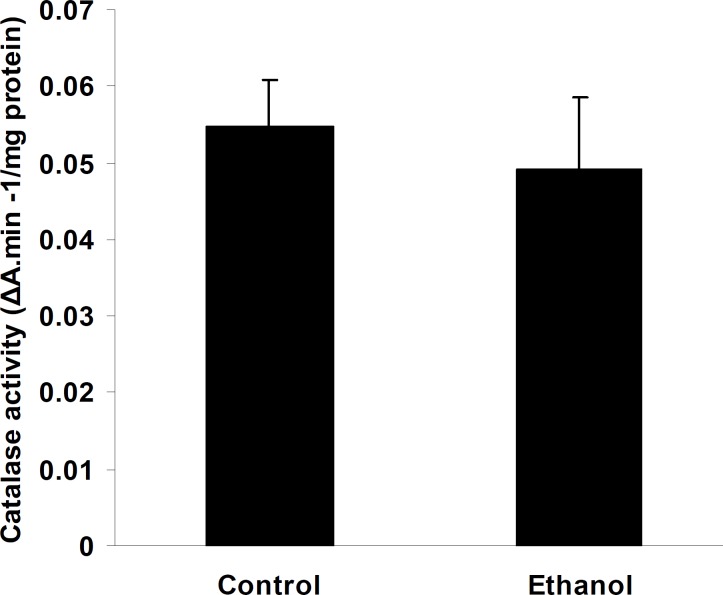
Catalase activity in the cerebellum of control and pups exposed to ethanol (6 g/kg) or a vehicle (NaCl 0.9%) intraperitoneal injection. CAT activity showed reduction in ethanol group as compared with control but was not significant. Results are presented as mean ± SEM (n=7 per group). ΔA: Absorption change.

**Figure 5:**
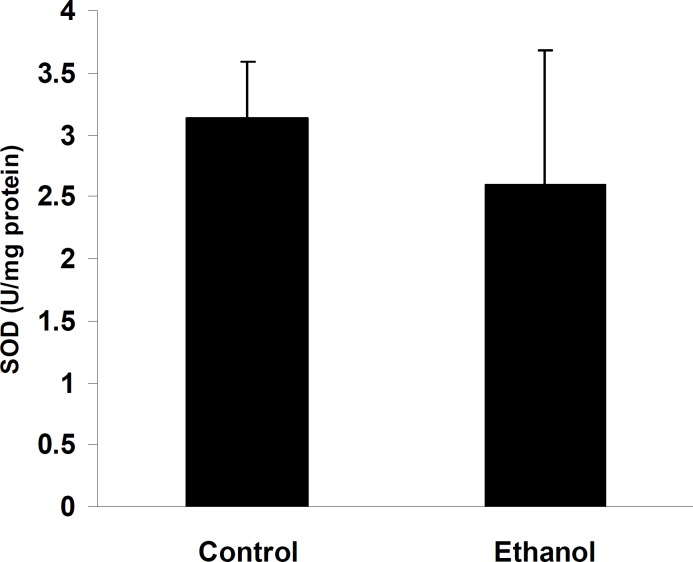
Superoxide dismutase activity in the cerebellum of control and pups exposed to ethanol (6 g/kg) or a vehicle (NaCl 0.9%) intraperitoneal injection. SOD activity showed reduction in ethanol group as compared with control but was not significant. Results are presented as mean ± SEM (n= 7 per group).

Administration of ethanol significantly increased TBARS levels in the cerebellum compared to control rats (0.36±0.09 in ethanol treated pups compared to 0.03±0.01 in control; Figure 2; *P*< 0.01, n=7). Pups treated with ethanol exhibited a marked decrease in the activities of GPx (0.38±0.006 in ethanol treated pups compared to 0.54±0.01 in control; Figure 3, *P*< 0.01, n=7), whereas, in spite of a reduction in the cerebellum CAT (0.054±0.006 and 0.049±0.009 in control and ethanol groups, respectively; Figure 4, n=7) and SOD activities (3.1±0.46 and 2.6±1.09 in control and ethanol groups, respectively; Figure 5, n=7) as compared to the control group, there were not significant differences. 

**Figure 6:**
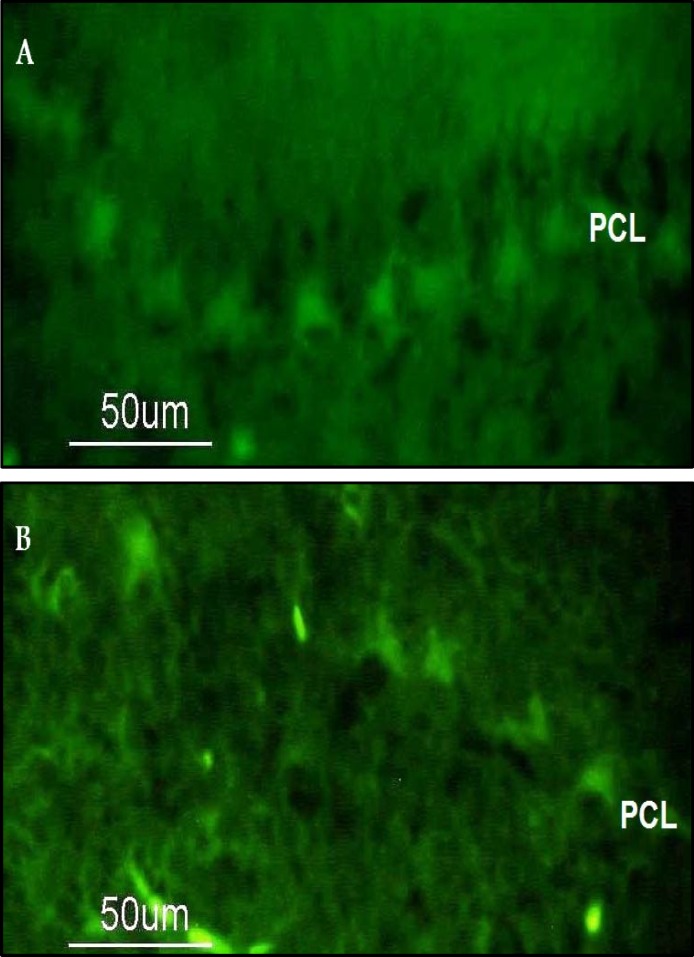
Photomicrograph showing Purkinje cell layer in lobule I of cerebellar vermis from P5 rat pups following exposure on P4–5 to (A) NaCl 0.9% or (B) ethanol (6 g/kg). Representative pictures obtained by immunostaining using antibody to calbindin D28k.

Histological sections of the vermis demonstrated changes in the Purkinje neurons. Cerebellar cortex immunohistochemistry showed relatively uniform structure. A typical three layer arrangement of neurons was noted (Molecular, Purkinje and Granular layers). However, some of the Purkinje cells of the ethanol group were sparse, shrunken, and irregularly shaped. Their Purkinje cell layers showed a disconnected pattern, with a wide gap between the cells (Compare Figure 6 A with B).

*** Significantly different from the control, *P*< 0.001

** Significantly different from the control, *P*< 0.01

** Significantly different from the control, *P*< 0.01

Note the relative depletion of Purkinje cells in the ethanol-exposed animal compared to the animal receiving the NaCl 0.9%.

M: Molecular layer; P: Purkinje cell layer; G: Granular cell layer

## Discussion

Epidemiological studies indicate that fetal alcohol syndrome is a major public health problem that concerns all industrialized countries (36). Neuronal vulnerability to ethanol coincides with a period of intense synaptogenesis that, in humans, starts during the third trimester of pregnancy (37). The first 10 postnatal days in the rat are commonly referred to as the ‘third-trimester equivalent’ period of brain development (38). Additionally, a defined period of Purkinje cell vulnerability occurs during PN4-6 in the rat during which the peak BEC determines the extent of Purkinje cell loss (10, 11, 13). However, the mechanisms underlying the deleterious effects of ethanol on the developing brain remain largely unknown and no efficient treatment is currently available. Our present findings demonstrate that administration of ethanol during the early postnatal period (PD 4-5) is able to induce widespread neurodegeneration in the cerebellum (loss of Purkinje cells in lobule I). The results were consistent with previous reports that Purkinje neurons in the rat cerebellum demonstrate cell death following ethanol exposure during postnatal days 4–6 (11, 13), the magnitude of which is correlated with blood ethanol levels (13). Goodlett and Eilers *et al* (10) and Light *et al* (12) have shown that alcohol exposure limited to PD 4, PD 4-5, or PD 4-6 has a long term effect on the final number of Purkinje cells quantified in the rat cerebellum at various ages, demonstrating a permanent effect of a brief alcohol treatment at this sensitive time window.

Previous studies have shown that the cerebellum to body weight ratios were diminished after ethanol administration (29, 39). Severity of alcohol induced reduction in total brain weight is dependent on dosage and duration of ethanol exposure (4, 40, 41). In our study, the cerebellum to body weight ratio were significantly (*P*< 0.001) diminished by ethanol exposure.

Oxidative stress is attractive as a possible mechanism for the alcohol-induced brain damage for many reasons. The brain processes large amounts of O_2_ in relatively small mass, and has a high content of substrates available for oxidation (i.e. polyunsaturated fatty acids and catecholamines) in conjunction with low antioxidant activities, making it extremely susceptible to oxidative damage (42). The developing brain, which has only a fraction of the antioxidant enzyme activity of the adult brain, is perhaps even more vulnerable to the neurotoxic effects of oxidative stress than the adult brain (42). 

In the *in vivo* cerebellum, reactive oxygen species are increased after ethanol exposure during the PN4–6 vulnerable window (43), and endogenous antioxidants increase following exposure outside the window (18). This finding that ethanol exposure during the vulnerable window failed to result in an increase in tissue antioxidant capacity has supported the approach of providing supplemental antioxidants to identify if the Purkinje neuron loss could be mitigated (25, 26, 44, 45).

In the current study, we found that ethanol significantly (*P*< 0.05) increased lipid peroxidation and decreased GPx activities (*P*< 0.05) in the rat cerebellum. Several studies have examined the role of oxidative stress in developmental alcohol-mediated neurotoxicity, possibly via the formation of free radicals (25, 46). Acute ethanol administration produces lipid peroxidation in the brain, as indicator of oxidative stress (47, 48). Increased oxidative stress occurs directly due to ethanol and its oxidation products (48). Ethanol is extensively metabolized into cytotoxic acetaldehyde by alcohol dehydrogenase enzyme in the liver. The acetaldehyde must be further oxidized to acetate by acetaldehyde dehydrogenase enzyme, which is present in the brain and is capable of producing reactive oxygen species (ROS) (49). It has been demonstrated that ethanol induces the synthesis of cytochrome P450 (CYP2E1) in the brain. CYP2E1 is present in various brain regions, and it may be an important source of ethanol induced oxidative stress (50). It also increases the NADH/ NAD ratio, which causes reduction of ferric iron to ferrous iron, a potent generator of the hydroxyl radical, which can then cause lipid peroxidation. The main damage to cells after ethanol results from the ROS-induced alteration of macromolecules, such as polyunsaturated fatty acids in membrane lipids, proteins, and DNA (47, 51). Lipid peroxidations, especially in membranes, play a crucial role in tissue injury (52).

Heaton *et al* demonstrated that neonatal alcohol exposure on postnatal days (PDs) 4 and 5 significantly reduced the Purkinje cell numbers in lobule I of the cerebellum and that such deficits were attenuated by pretreatment and cotreatment with vitamin E (25). Also, Shirpoor *et al* demonstrated that oxidative stress plays a crucial role in alcohol-induced brain damage, mainly by induction of apoptosis and administration of vitamin E in gestation and lactation periods alleviate oxidative stress, via decreasing protein oxidation and lipid peroxidation (53).

GPx is the most important antioxidative enzymes in the brain that metabolizes peroxides such as H_2_O_2_ protects cell membranes from lipid peroxidation. McCary *et al* indicated that glutathione peroxidase activity inhibits lipid peroxidation in membrane, as glutathione peroxidase activity exert its effect on this system by preventing free radical attack on the polyunsaturated membrane lipids in the first place (54). Therefore, it seems that ethanol could through decreasing GPx activity result in lipid peroxidation and Purkijne cell loss.

Both human and animal research provide evidence that the CNS is vulnerable to the damaging effects of ethanol during development, and one particular form of damage is neuronal loss. Alcohol exposure during the period of extensive connectivity among Purkinje and other neurons, either from postnatal days (PD) 4-6 (10-13) or only on PD 4 (5), results in permanent loss of Purkinje cells. The consequence of Purkinje cell loss are thought to involved in the deficiencies of motor coordination and gait exhibited by children diagnosed with fetal alcohol syndrome (55).

## Conclusions

Ethanol exposure during the vulnerable window results in loss of cerebellar Purkinje cells. Purkinje cell loss is, in part through decrease in the activities of GPx and increase of lipid peroxidation in the rat cerebellum. 
